# The Important Role of Halogen Bond in Substrate Selectivity of Enzymatic Catalysis

**DOI:** 10.1038/srep34750

**Published:** 2016-10-06

**Authors:** Shuiqin Jiang, Lujia Zhang, Dongbin Cui, Zhiqiang Yao, Bei Gao, Jinping Lin, Dongzhi Wei

**Affiliations:** 1 State Key Laboratory of Bioreactor Engineering, New World Institute of Biotechnology, East China University of Science and Technology, Shanghai 200237, China

## Abstract

The use of halogen bond is widespread in drug discovery, design, and clinical trials, but is overlooked in drug biosynthesis. Here, the role of halogen bond in the nitrilase-catalyzed synthesis of ortho-, meta-, and para-chlorophenylacetic acid was investigated. Different distributions of halogen bond induced changes of substrate binding conformation and affected substrate selectivity. By engineering the halogen interaction, the substrate selectivity of the enzyme changed, with the implication that halogen bond plays an important role in biosynthesis and should be used as an efficient and reliable tool in enzymatic drug synthesis.

Halogens (X = F, Cl, Br, and I), as pharmaceutically active ligand substituents, are widely used in pharmacology^1^,^2^. Approximately 50% molecules in high-throughput screening are halogenated^1^ and around 40% drugs currently on the market or in clinical trials are halogenated^3^. Furthermore, an estimated 25% medicinal chemistry papers and patents involve the addition of halogen atoms at a late stage of the synthesis^1^. Halogens, treated primarily as electron-rich atoms that do not participate in specific interactions^4^, form a halogen bond (X-bond) with a proximal halogen-bond acceptor (such as O, N, S, and aromatic ring)^5^,^6^,^7^,^8^. The halogen bond, analogous to the hydrogen bond, is a highly directional and specific non-covalent interaction^9^. This bond has attracted great attention in pharmacology because halogen bonds, as orthogonal molecular interactions to hydrogen bonds, can be introduced to improve ligand affinities without disrupting other structurally important interactions^10^, and thus can be exploited for the rational design of halogenated ligands as inhibitors and drugs^11^.

The halogen bond, which has a wide application in the pharmaceutical sector, including drug discovery, design, and clinical trials, has been nonetheless overlooked in enzymatic catalysis, generally regarded as a practical and environmentally-friendly alternative to the traditional metallo- and organocatalysis in drug synthesis^12^. Nevertheless, the halogen bond is also popular in protein-ligand complexes, with >1000 structures in 2010 and >2000 in recent years^13^. Regardless, the importance or prevalence of the halogen bond in the biosynthesis of drugs or drug precursors remains unclear.

Nitrilase (EC 3.5.5.1), catalyzing the hydrolysis of nitriles to the corresponding acids in a single step reaction^14^, plays an important role in the manufacture of key building blocks for drugs, such as clopidogrel^15^, atorvastatin (Lipitor)^16^ and pregabalin^17^. This not only on account of the mild reaction conditions but also because of the regioselectivity and enantioselectivity of the nitrilase^18^. Each isomer of ortho-, meta-, and para-halogenated precursors or drugs should be used individually because of the specific pharmaceutical activity. For example, ortho-chlorophenylacetic acid can be used to synthesize diclofenac^19^ and clopidogrel^20^, an anti-inflammatory drug and anti-platelet aggregation drug, respectively; para-chlorophenylacetic acid can be used to synthesize indoxacarb^21^ and baclofen^22^, an insecticide and a muscle relaxer for treating muscle symptoms caused by multiple sclerosis, respectively. However, naturally occurring nitrilase is characterized mainly by meta-activity, seldom by para-activity, but not ortho-activity^23^. Therefore, it is crucial to engineer nitrilase substrate selectivity for each isomer of the ortho-, meta-, and para-halogenated compounds.

In this study, we undertook the design of nitrilase enzymes with altered specificities for substrate isomers. We used mutagenesis to specify potential halogen bonding interactions with the chloro-substituents at ortho-, meta-, or para-positions (Fig. 1A). We started by analyzing the active site of the wild type enzyme and, after performing molecular dynamics (MD) simulations, we designed mutants in the substrate binding pocket to engineer X-bonds between the substrate and protein side-chains. Thus, enzyme substrate specificity was directed towards one or more of the isomeric forms. The results of this study demonstrate the potential for exploiting X-bonds as a recognition element in protein engineering, particularly in helping to define and alter the specificity of enzymes in their catalytic site. Our study shed light on the role of halogen bonds in drug biosynthesis and suggests that more attention should be paid to the application of the halogen bond in enzymatic synthesis of drugs in the future.

## Results

Nitrilase from *Syechocystis* sp. PCC6803, whose structure has been reported in our previous work (PDBID: 3WUY)^24^, exhibited high selectivity for meta-chlorobenzyl cyanide (**1a**) but not para-chlorobenzyl cyanide (**1b**) (Table 1). The difference between **1a** and **1b** concerns just the location of the halogen atom, Cl, which can form halogen bond with the proximal halogen-bond acceptor. The halogen bonds in the two complexes were carefully analyzed by a 10-ns MD simulation (Supporting Information Figure S1). In the nitrilase WT–**1a** complex, a halogen bond was formed between **1a** and Gly195, while it was formed with Tyr173 for WT–**1b** complex. The different halogen atom location caused a distinct distribution of the halogen bond. Because the interactions between the substrates and the enzyme contribute to the conformation of the substrate-enzyme conformations^25^,^26^, this changing distribution of the halogen bond may result in a distinct substrate binding mode, greatly contributing to the substrate selectivity^27^.

**Figure 1:**
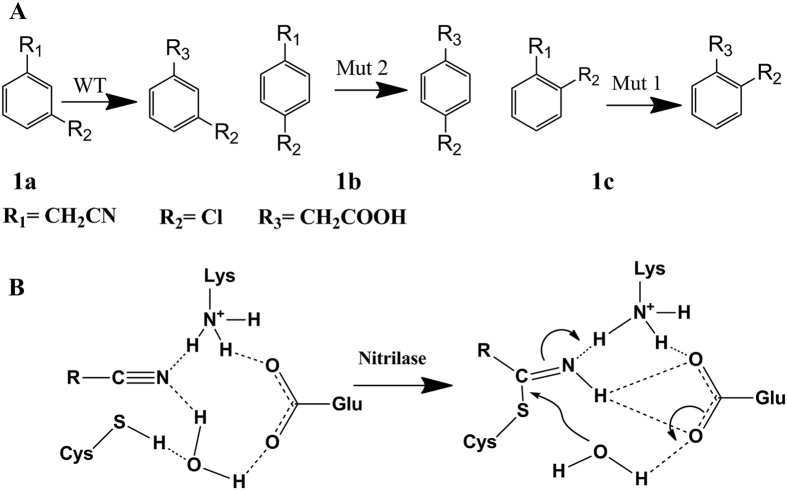
(**A**) The nitrilase substrate selectivity of ortho-, meta-, and para-isomers, (**B**) Proposed nitrilase reaction mechanism.

**Figure 2:**
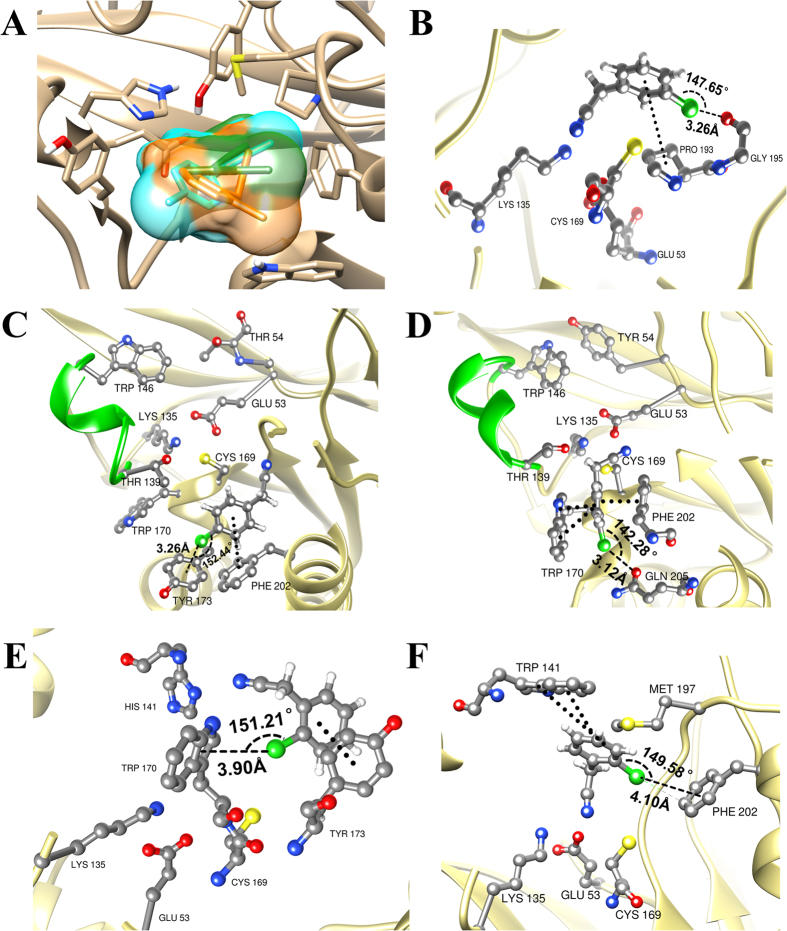
The surface of **1a**, **1b** and **1c** in the binding pocket of WT. **1a**, **1b** and **1c** are shown in green, cyan and orange (**A**). And the halogen bonds and aromatic interactions in complexes: WT-**1a** (**B**), WT-**1b** (**C**), T54Y-**1b** (**D**), WT-**1c** (**E**), H141W-**1c** (**F**). Key residues are presented as ball and stick with carbon atoms in gray, hydrogen in white, oxygen in red, nitrogen in blue, chloride in green, sulfur in yellow. And loop 139–140 was presented as cartoon in green. The geometric information of halogen bonds in this figure can be accessed in Supporting Information Table S3.

Careful analysis of the binding states of the two substrates was performed. First, no steric clashes were apparent for **1b** because of the large binding pocket of this nitrilase (Fig. 2A). According to the catalytic mechanism^28^ of the nitrilase superfamily (Fig. 1B), the nitrilase utilizes the Glu53–Lys135–Cys169 catalytic triad to hydrolyze non-peptide carbon-nitrogen bonds. The first and decisive step is a nucleophilic attack on a cyano or carbonyl carbon atom by the active site cysteine residue. D_C8-SG_ describes the distance from the C8 carbonyl carbon atom of the substrate to the mercapto sulfur SG of the catalytic cysteine, which determines whether the nucleophilic attack can occur (Fig. 1B). Meanwhile, the lysine formed hydrogen bonds with the substrate to provide stabilization for the intermediate. D_N1-HZ_ is the distance between the N1 cyano nitrogen of the substrate and HZ of the catalytic lysine, which affects the stabilization of the substrates (Fig. 1B). Therefore, shorter D_C8-SG_ and D_N1-HZ_ both indicate a greater probability of the substrate being catalyzed. A 10-ns MD simulation of the two complexes (WT-**1a** and WT-**1b**) indicated that for this nitrilase, the average D_C8-SG_ and D_N1-HZ_ values with **1a** were 3.76 Å and 2.83 Å, respectively; and those with **1b** were 6.54 Å and 9.49 Å, respectively. The binding conformation of **1a** facilitated the nucleophilic attack and the formation of transition state. This was not the case for the binding conformation of **1b**. Therefore, for WT enzyme, the orientation of the cyano group of **1a** toward the catalytic triad was stabilized by the halogen bond between **1a** and Gly195 and the hydrophobic stacking interaction between **1a** and Pro193 (Fig. 2B). Meanwhile, in the WT–**1b** complex, the main halogen bond was formed between **1b** and Tyr173. Because the triple-bond between the carbon and nitrogen atoms and the phenyl ring are structurally rigid, the direction change on one side of the phenyl ring, such as para-Cl, would cause a corresponding direction change on the other side, such as a triple-bond of carbon and nitrogen. Thus, the direction change of para-Cl caused the cyano group to position away from the catalytic triad, which was further retained by an perpendicular aromatic interactions formed between Phe202 and **1b** (Fig. 2C).

Hence, the halogen bonds, together with aromatic interactions, affected substrate selectivity of WT for **1a** and **1b**, with the halogen bonds serving as a crowbar and the aromatic interactions controlling the rotation of substrates. These interactions may be maintaining the cyano group in the proximity of the catalytic triad, and positioning it in an orientation favorable for the enzymatic reaction to occur. It seems to be feasible that the modulation of the halogen bonds would affect enzyme substrate selectivity. However, a direct and precise modulation is rather difficult since the halogen bond, like the hydrogen bond, is a complex network of interactions. Here, a simple protocol was designed to model modulation of the halogen bond. First, residues in the substrate binding pocket^24^ were selected to be mutated to aromatic amino acids (Phe, Tyr, Trp), since the binding pocket is “aromatic rich” and aromatic amino acids are involved in halogen bond^7^,^8^. The mutants were constructed by *in silico* mutation. Second, enzyme-substrate complex conformations, reflecting both interactions^25^,^26^ and catalytic property^27^, were used to screen the shifted selectivity mutants. The screening criteria were that both D_C8-SG_ and D_N1-HZ_ should be shorter than the corresponding distances in WT. MD Simulation (10-ns MD) was performed to analyze the enzyme-substrate complex conformations. Finally, mutagenesis and catalytic experiments with the screened mutants were performed *in vitro*.

With **1b**, T54Y mutant D_C8-SG_ and D_N1-HZ_ distances were shorter compared with WT (Supporting Information Table S1). Next, 10-ns MD simulation was performed to investigate the dynamic conformation of T54Y (Supporting Information Figure S2). The productive binding conformation involved a T54Y–**1b** complex with small and stable D_C8-SG_ and D_N1-HZ_ (average values of 4.31 Å and 7.90 Å, respectively), while those with **1a** bound to T54Y were relatively large (average values of 5.74 Å and 8.54 Å, respectively). In addition, the distribution of halogen bond in T54Y-**1a** and T54Y-**1b** was changed compared with WT. In the T54Y–**1b** complex, the halogen bond was formed between **1b** and Gln205. Additionally, when Thr54 was mutated to Tyr, the increased steric occupation pushed the loop of residues 139–146 back, away from the center of the binding pocket, making space for the side chain of Trp170. Further, **1b** formed π-π stack interaction with Trp170 and Phe202, pulling the cyano group of **1b** toward the catalytic triad (Fig. 2D). On the other hand, in the T54Y–**1a** complex, **1a** formed halogen bond with Tyr173 and an aromatic interaction with Phe202, with both keeping the cyano group away from the catalytic triad. Hence, the modulation of the halogen bonds, together with the aromatic interactions, manipulated the substrate binding conformation. Binding conformations of T54Y with **1a** and **1b** suggested enzyme substrate selectivity for **1b**. Hence, T54Y was selected for testing. As expected, *K*_cat_K_M_^−1^ analysis revealed that the catalytic efficiency of T54Y with **1b**, 0.56 s^−1^ mM^−1^, was much higher than that with **1a**, 0.01 s^−1^ mM^−1^ (Table 1). The activity of T54Y with ortho-chlorobenzyl cyanide (**1c**) was also tested, but it was below the detection limit. We thus verified that the halogen bonds formed between the substrates and the enzyme strongly affect enzymatic substrate specificity and can be employed to shift substrate selectivity.

Furthermore, since few known natural nitrilases display ortho-selective activity, it is more challenging and valuable to shift the substrate selectivity to ortho-selectivity. Here, the same protocol was applied to design mutants with substrate selectivity for ortho-chlorobenzyl cyanide (**1c**) as above. No steric clashes were apparent for **1c** binding because of the large binding pocket of this nitrilase (Fig. 2A). The only difference between **1a** and **1c** was the halogen bond. In the WT–**1c** complex, the chlorine atom of **1c** formed a halogen bond with Trp170. A 10-ns MD simulation of this complex yielded average D_C8-SG_ and D_N1-HZ_ values of 7.60 Å and 9.06 Å, respectively. They were both larger than those of **1a**. Therefore, the binding conformation of **1c** was not conducive to nucleophilic attack and transition state formation, because the halogen bond formed between ortho-chlorine atom and Trp170, and the perpendicular aromatic interactions between substrate and Trp173, cooperatively kept **1c** cyano group away from the catalytic triad (Fig. 2E). We constructed and analyzed the mutants, as described above, and *in silico* screened their interaction with **1c**.

With **1c**, H141W mutant D_C8-SG_ and D_N1-HZ_ distances were short, indicating a potential productive conformation (Supporting Information Table S1). Additionally, the dynamic conformation of H141W complexed with **1c** and **1a** was analyzed by a 10-ns MD simulation. Distances D_C8-SG_ and D_N1-HZ_ of H141W–**1c** complex remained small and stable (average values of 3.79 Å and 3.46 Å, respectively), while those with **1a** were not only large but also variable (average values of 10.40 Å and 14.76 Å, respectively; Supporting Information Figure S2). These results of conformational analyses demonstrated a potential substrate selectively of H141W toward **1c**. This binding mode change may stem from the rearrangement of the halogen bond. As expected, in the H141W–**1c** complex, the halogen bond was mainly formed with Phe202 (80%), positioning the CN group to the active site, with the assistance of an aromatic interaction with Trp141 (Fig. 2F). On the other hand, for **1a**, the main halogen bond formed with Ile201 (95.08%) and a sigma-hole…π interaction arose with Phe202, cooperatively placing the cyano group away from the catalytic triad. Therefore, H141W was selected to testing. As anticipated, the H141W mutant, which showed a productive binding conformation with **1c**, was really highly selective for **1c**, according to the *K*_cat_K_M_^−1^ analysis (0.33 s^−1^ mM^−1^, Table 1). The catalytic efficiency of the mutant was below the detection limit, for both **1a** and **1b**. Hence, the data once again confirmed that the halogen bonds, working as a crowbar for the cyano group, together with the aromatic interactions, strongly affect substrate selectivity in biosynthesis.

Additionally, the putative X-bonding substituent Cl was varied to methyl group at each isomer position to futher evidence that the Cl in any of the isomeric forms participate in X-bonding in this enzyme system. From the experiment results (Supporting Information Table S2),wild-type showed activity to both meta-, and para-methylbenzyl cyanide (relative acitvity of 100% and 46.2%,respectively), and H141W and T54Y both showed selectivity to meta-methylbenzyl cyanide (relative acitvity of 27.18% and 20.54%, respectively). The H141W and T54Y showed similar selectivity with wild-type. The resulting isomer activity may come from steric or hydrophobic effects since the methyl substituent does not involve in halogen bond. Compared with methyl substituent, when the substituent is Cl, wild-type showed more rigid substrate selectivity to meta-isomer, indicating the halogen bond enhanced the substrate selectivity. Besides, wild-type, H141W and T54Y exhibited the different substrate selectivity to chlorobenzyl cyanide, but the same s pecificity to meta-methylbenzyl cyanide, indicating that the halogen bond participated in the substrate selectivity of the enzymes. Therefore, the engineered enzymes actually utilized X-bonding as the driver of substrate specificity in this study.

## Discussion

In conclusion, the role of halogen bond in nitrilase substrate selectivity was herein investigated. Detailed conformational analysis revealed that the halogen bond induced a change of substrate binding mode, serving as a crowbar, and the aromatic interactions controlled the positioning of the benzene ring of the substrate. Together, the bonds held the substrate cyano group in proximity to the catalytic triad, and positioning it in a reaction-favoring orientation. Furthermore, by regulating the halogen bond, two types of artificial enzymes were obtained, with selective activity toward para-chlorobenzyl cyanide and ortho-chlorobenzyl cyanide, respectively. Therefore, halogen bond formed between the substrate and the nitrilase strongly affected substrate selectivity of the enzyme.

Besides, the current study assessed the performance of standard Amber ff99SB force field (Supporting Information Table S4 and Figures S4–S6) and the positive extra-point (PEP) modified one in describing the halogen bond. In the PEP approach^29^,^30^, the σ-hole on the halogen atom is represented by an extra-point of positive charge. From the MD results of PEP, the different distribution of halogen bond contributes majorly to the substrate selectivity of wild-type. Also H141W showed shorter D_C8-SG_ and D_N1-HZ_ to **1c** and T54Y exhibited both shorter key distances to **1b**, indicating H141W and T54Y were selectively active to **1c** and **1b**, respectively. Compared with the standard form Amber force field, the residues paticipating in X-bond were more focused in PEP, indicating the X-bonds were enhanced with EP integrated. Therefore, the modified Amber force field such as PEP is more accurate in describing the property of X-bonds.

Generally, halogen bonds formed between proteins and their ligands have been widely employed in drug design because of their role in improving ligand binding affinities. However, enzymatic catalysis, which is generally regarded as a practical and-environmentally friendly approach to drug synthesis, has ignored the halogen bond. This study demonstrated for the first time that halogen bond significantly affects substrate binding conformation and, further, plays an important role in substrate selectivity. Therefore, in a protein-ligand biosystem, the halogen bond can shift the enzyme’s substrate selectivity by adjusting the substrate binding conformation. It can be used as a powerful tool, similarly to hydrogen bond^7^,^8^,^31^,^32^ and electrostatic interactions^33^ in enzyme design. Consequently, the halogen bond in the biocatalysis cannot be ignored and more attention should be paid to it.

## Methods

The expression and purification of nitrilase and the enzyme assay were conducted as described previously^15^,^24^,^34^, while an eluting solvent system was phosphoric acid (0.1%, v/v) and methanol (40:60, v/v).

### Computational Details

**Figure 3:**
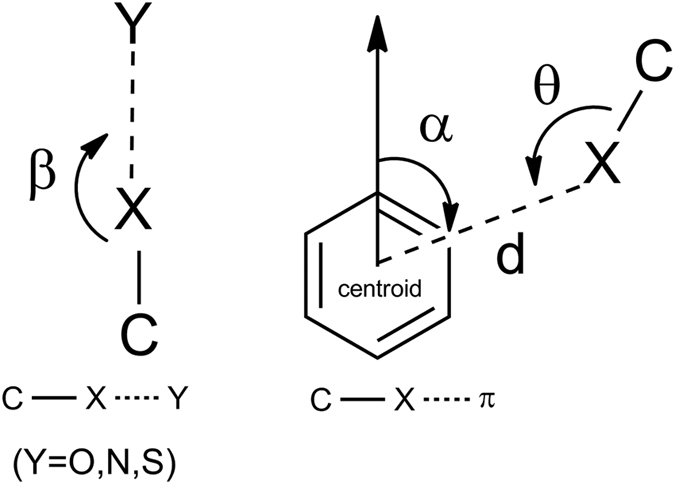
Geometric model of two types of halogen bond, C–X···Y and C–X···π.

The initial structure of the nitrilase was crystal structure (PDBID: 3WUY) obtained in our previous work. All of the molecular docking experiments and virtual mutation utilized here were performed on Discovery Studio 4.0^35^. Default parameters were used in our docking experiments. The substrate conformation with highest score calculated by Discovery Studio 4.0 was selected for MD simulation. The substrates halogenated by chlorine were constructed and their geometries were optimized at the HF/6-31G* level by Gaussian 03. The atomic partial charges were then evaluated with incorporating an extra point of charge on each isomer’s halogen atom using the restrained electrostatic potential (RESP) approach^36^. The distance between the halogen and the extra point is 1.90 Å^37^. The parameters used for the extra point were set as described by Ibrahim^38^. The Amber ff99SB force field^39^ was applied to treat the proteins, and the Generalized Amber Force Field (GAFF)^40^ was employed to deal with the substrates. The complex was solvated in a TIP3P water box, and sodium ions were added to ensure the electric neutrality. MD simulation was carefully performed with Amber14^41^,^42^ in 7 stages. The system was minimized with position restraints of 50.0 kcal mol^−1^ Å^−2^ and 20.0 kcal mol^−1^ Å^−2^ in the first and second stages, respectively, and with no restraint in the third stage. The minimization for each stage took 20,000 steps to reach convergence. Then, the system was heated from 0 K to 300 K in 0.05 ns with a restraint of 10.0 kcal mol^−1^ Å^−2^ using the Andersen temperature coupling scheme^43^. The next stage was the equilibration with a restraint of 2.0 kcal mol^−1^ Å^−2^ for 0.05 ns, followed by full system equilibration with no restraints for 0.5 ns. Finally, the MD simulation was performed at 300 K for 10 ns. Non-bonding interactions were calculated using a cutoff of 14 Å. The SHAKE algorithm^44^ was employed to restrain all bonds involving hydrogen atoms. Langevin dynamics^45^ was applied to regulate the temperature with a collision frequency of 2.0 ps^−1^. The pressure was controlled using the isotropic position scaling protocol. All MD simulations were performed using the AMBER14 program. The criteria of halogen bond was set according to Weiliang Zhu *et al*.^3^,^13^, as X···Y distances shorter than the sum of vdW radii, (d(Cl···O) < 3.27 Å, d(Cl···N) < 3.30 Å, d(Cl···S) < 3.55 Å, the C–X···Y angle β is larger than 140°. For C–X···π halogen bond, π systems from aromatic residues (Phe, Tyr, His, and Trp) are considered in this study with the following criteria: d(Cl···π) < 4.2 Å, α < 60°, and θ > 146° (Fig. 3).

## Additional Information

**How to cite this article**: Jiang, S. *et al*. The Important Role of Halogen Bond in Substrate Selectivity of Enzymatic Catalysis. *Sci. Rep*. **6**, 34750; doi: 10.1038/srep34750 (2016).

## Supplementary Material

Supplementary Information

## Figures and Tables

**Table 1:** Comparison of the kinetic constants and key distances obtained for the wild-type and mutant nitrilase.

Enzyme	Substrate	K_M_ (mM)	K_cat_ (s^−1^)	K_cat_K_M_^−1^ (s^−1^mM^−1^)	D_C8-SG_^b^ (Å)	D_N1-HZ_^b^ (Å)
**WT**	**1a**	0.46 ± 0.04	0.27 ± 0.02	0.59	3.76 ± 0.21	2.83 ± 0.56
	**1b**	NA^a^	NA^a^	NA^a^	6.54 ± 0.33	9.49 ± 0.52
	**1c**	NA^a^	NA^a^	NA^a^	7.60 ± 0.58	9.06 ± 1.19
**H141W**	**1a**	NA^a^	NA^a^	NA^a^	10.40 ± 2.13	14.76 ± 2.84
	**1b**	NA^a^	NA^a^	NA^a^	10.27 ± 1.62	10.13 ± 2.08
	**1c**	0.65 ± 0.18	0.21 ± 0.05	0.33	3.79 ± 0.21	3.46 ± 0.98
**T54Y**	**1a**	9.60 ± 0.27	0.08 ± 0.13	0.01	5.74 ± 0.81	8.54 ± 0.99
	**1b**	0.71 ± 0.03	0.56 ± 0.07	0.56	4.31 ± 0.50	7.90 ± 0.75
	**1c**	NA^a^	NA^a^	NA^a^	6.16 ± 0.44	10.64 ± 0.76

D_C8-SG_ is the distance between the SG in Cys169 of the nitrilase and cyano group (C8) in substrates. D_N1-HZ_ is the distance between the HZ atom in Lys135 of the nitrilase and cyano group (N1) in substrates.

^a^Activity below the detection limit.

^b^The average value during the 10 ns molecular dynamics performed using the extra-point modified AMBER99 force field. Data are reported as mean ± standard deviation of three independent experiments and that of all 1000 frames generated from the 10 ns molecular dynamics.
