# Supplementary material for: The Important Role of Halogen Bond in Substrate Selectivity of Enzymatic Catalysis
Source: Sci Rep. 2016 Oct 6;6:34750. doi: 10.1038/srep34750 (PMC5052520; doi:10.1038/srep34750)
Supplement: Supplementary Information [file srep34750-s1.pdf]

Supporting Information

The Important Role of Halogen Bond in Substrate Selectivity of Enzymatic Catalysis

Shuiqin Jiang, Lujia Zhang*, Dongbing Cui , Zhiqiang Yao, Bei Gao, Jinpin Lin and Dongzhi Wei*

Correspondence may be addressed to either of these authors (Dongzhi Wei, e-mail: dzhwei@ecust.edu.cn or Lujia Zhang, e-mail: ljzhang@ecust.edu.cn)

Table S1. D_{N1-HZ} and D_{C8-SG} in the complexes of the nitrilase virtual mutations with substrate 1b (A) and 1c (B)

A

	WT	**T54Y**	T54W	M197F	M197Y	M197W
D_{N1-HZ}(Å)	2.845	**2.442**	2.707	2.874	3.362	3.009
D_{C8-SG}(Å)	3.555	**3.398**	3.746	4.328	4.137	4.234
	S63F	S63Y	S63W	F64Y	F64W	T137F
D_{N1-HZ}(Å)	3.335	3.302	2.955	3.347	3.356	2.891
D_{C8-SG}(Å)	3.574	3.573	3.770	3.815	3.859	3.586
	T137Y	T137W	P138F	P138Y	P138W	T139F
D_{N1-HZ}(Å)	3.985	3.662	2.976	2.888	2.911	3.147
D_{C8-SG}(Å)	3.868	3.474	3.631	3.758	3.729	3.512
	T139Y	T139W	Y140F	Y140W	H141F	H141Y
D_{N1-HZ}(Å)	3.388	6.027	3.040	3.123	2.883	3.057
D_{C8-SG}(Å)	3.483	4.376	3.605	3.501	3.623	4.013
	H141W	R143F	R143Y	R143W	A168F	A168Y
D_{N1-HZ}(Å)	2.925	2.900	2.928	2.945	2.939	1.965
D_{C8-SG}(Å)	3.756	3.841	3.583	3.955	4.109	4.114
	A168W	W170F	W170Y	E171F	E171Y	E171W
D_{N1-HZ}(Å)	4.058	2.883	2.972	3.010	3.538	3.522
D_{C8-SG}(Å)	4.366	3.904	3.752	4.399	4.130	4.258
	Q192F	Q192Y	Q192W	F193Y	F193W	P194F
D_{N1-HZ}(Å)	3.007	3.110	3.369	3.148	3.074	3.915
D_{C8-SG}(Å)	3.798	4.206	3.855	4.327	3.838	3.450
	P194Y	P194W	F202Y	F202W	T54F	
D_{N1-HZ}(Å)	3.130	3.384	3.018	3.407	3.368	
D_{C8-SG}(Å)	3.892	3.883	3.906	4.362	3.816	

B

	**WT**	T54Y	T54W	M197F	P194Y	P194W
$D_{N1\text{-}HZ}$(Å)	**2.906**	3.707	3.326	2.869	4.279	6.327
$D_{C8\text{-}SG}$(Å)	**3.256**	3.343	3.608	3.754	3.465	3.920
	S63F	S63Y	S63W	F64Y	F64W	T137F
$D_{N1\text{-}HZ}$(Å)	3.244	3.416	3.053	3.189	3.268	2.955
$D_{C8\text{-}SG}$(Å)	3.499	3.667	3.719	3.584	3.418	3.441
	T137Y	T137W	P138F	P138Y	P138W	T139F
$D_{N1\text{-}HZ}$(Å)	3.032	2.926	2.709	2.991	3.039	3.239
$D_{C8\text{-}SG}$(Å)	3.415	3.567	3.765	3.847	3.845	3.557
	T139Y	T139W	Y140F	Y140W	H141F	H141Y
$D_{N1\text{-}HZ}$(Å)	3.364	6.294	3.270	3.162	3.468	3.375
$D_{C8\text{-}SG}$(Å)	3.383	5.136	3.739	3.570	3.981	3.698
	**H141W**	R143F	R143Y	R143W	A168F	A168Y
$D_{N1\text{-}HZ}$(Å)	**2.540**	3.130	3.257	3.051	2.943	2.350
$D_{C8\text{-}SG}$(Å)	**3.132**	3.528	3.214	3.808	3.567	3.366
	A168W	W170F	W170Y	E171F	E171Y	E171W
$D_{N1\text{-}HZ}$(Å)	2.773	2.975	3.085	2.092	2.145	2.387
$D_{C8\text{-}SG}$(Å)	3.630	3.541	3.857	3.430	3.557	3.762
	Q192F	Q192Y	Q192W	F193Y	F193W	P194F
$D_{N1\text{-}HZ}$(Å)	2.960	2.929	3.183	2.971	3.381	4.505
$D_{C8\text{-}SG}$(Å)	3.518	4.123	3.802	3.342	3.892	3.430
	M197Y	M197W	F202Y	F202W	T54F	
$D_{N1\text{-}HZ}$(Å)	3.181	2.581	2.898	3.066	3.339	
$D_{C8\text{-}SG}$(Å)	3.896	4.065	3.577	3.125	3.385	

The bold entries represent the mutations which were implemented in wet experiment. $D_{N1\text{-}HZ}$ is the distance (in Å) between the HZ atom in Lys135 of the nitrilase and cyano group (N1) in substrates. $D_{C8\text{-}SG}$ is the distance (in Å) between the SG in Cys169 of the nitrilase and cyano group (C8) in substrates.

Table S2. The relative activity of the nitrilase wild-type and mutant enzymes to ortho-, meta-, and para-methylbenzyl cyanide substrates

Enzyme	Substrate	Relative activity (%)
**WT**	2a	100±5.17
	2b	46.20±6.18
	2c	NA[a]
**H141W**	2a	27.18±2.99
	2b	NA[a]
	2c	NA[a]
**T54Y**	2a	20.54±0.57
	2b	NA[a]
	2c	NA[a]

2a: meta-methylbenzyl cyanide, 2b: para-methylbenzyl cyanide, 2c: ortho-methylbenzyl cyanide, [a] Activity below the detection limit. Data are reported as mean ±standard deviation of three independent experiments.

Table S3. The geometric information of halogen bonds in figure 2

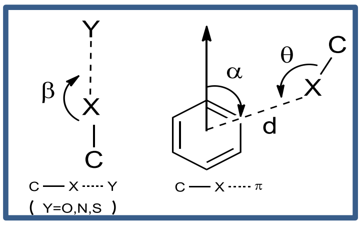

**Figure number**	**complex**	**X-bond acceptor**	d **(Å)**	β（°）	θ（°）	α（°）
2B	WT-1a	Gly195-O (back bond)	3.26	147.65		
2C	WT-1b	Tyr173 (aromatic side chain)	3.26		152.44	28.02
2D	T54Y-1b	Gln205-O (back bond)	3.12	142.28		
2E	WT-1c	Trp170 (aromatic side chain)	3.90		151.21	42.19
2F	H141W-1c	Phe202 (aromatic side chain)	4.10		149.58	43.21

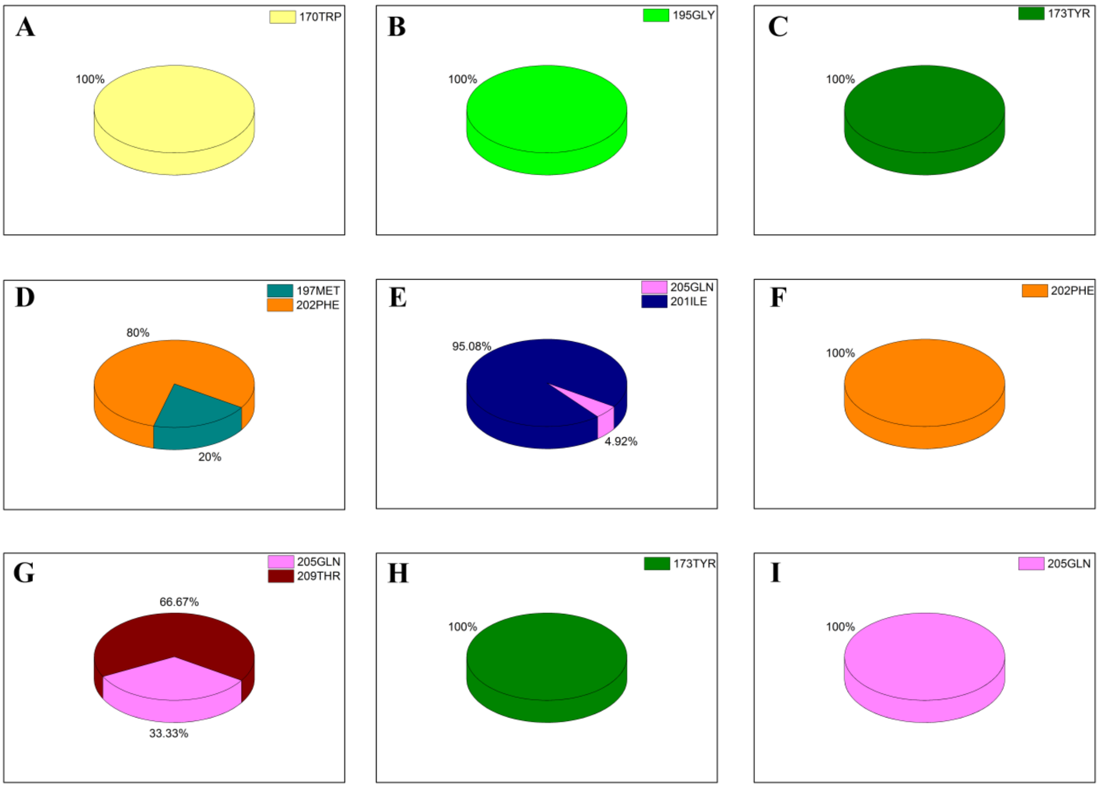

Figure S1. The profile of halogen bonds formed between substrates and different residues in the nitrilase and mutants during the 10 ns dynamic simulation performed using the extra-point modified AMBER99 force field. (A) WT and 1c (B) WT and 1a; (C) WT and 1b (D) H141W and 1c (E) H141W and 1a; (F) H141W and 1b (G) T54Y and 1c (H) T54Y and 1a; (I) T54Y and 1b

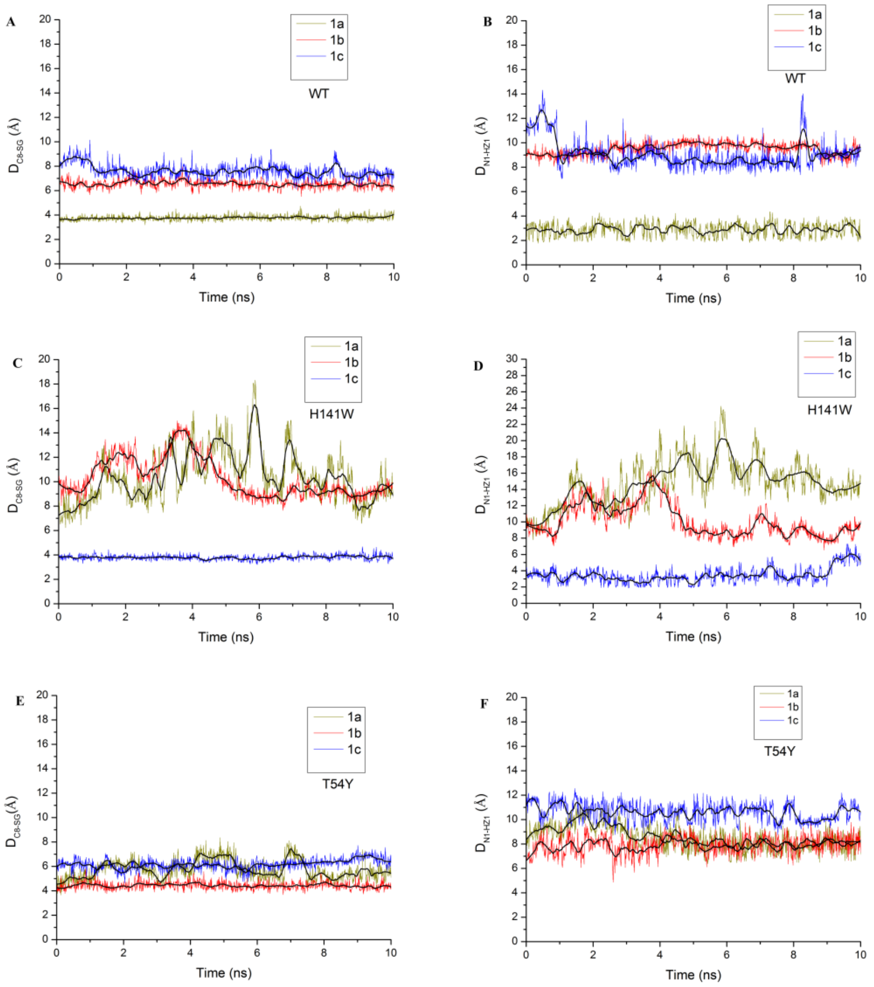

Figure S2. The $D_{N1\text{-}HZ1}$ and $D_{C8\text{-}SG}$ in the complexes of WT, H141W and T54Y with 1a, 1c and 1b during 10 ns MD simulation performed using the extra-point modified AMBER99 force field.

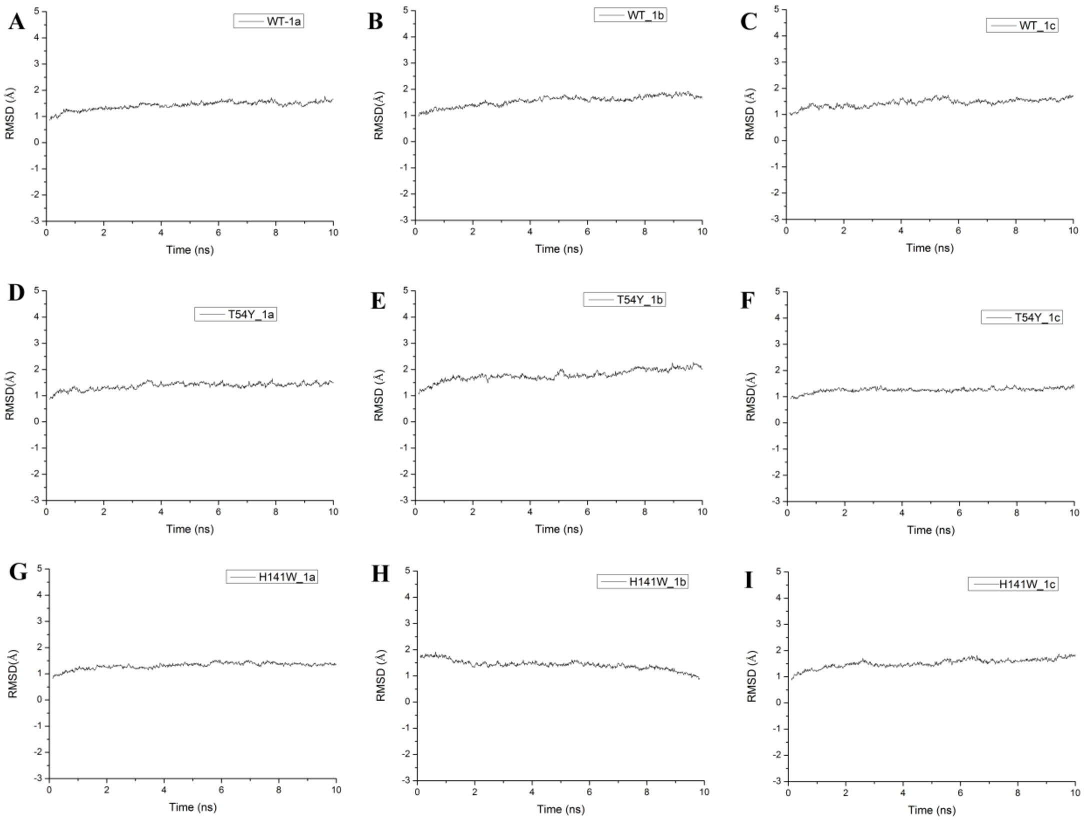

Figure S3. RMSD of the complexes of WT, H141W and T54Y with 1a, 1c and 1b during 10 ns MD simulation performed using the extra-point modified AMBER99 force field..

Table S4 Comparison of key distances obtained for the Nit6803 wild-type and mutant enzymes

**Enzyme**	**Substrate**	**$D_{C8\text{-}SG}$[b] (Å)**	**$D_{N1\text{-}HZ}$[b] (Å)**
**WT**	1a	3.88	3.57
	1b	5.20	8.67
	1c	5.97	9.86
**H141W**	1a	7.57	11.95
	1b	9.44	12.98
	1c	3.84	3.14
**T54Y**	1a	5.38	7.92
	1b	4.45	7.35
	1c	9.03	12.40

$D_{C8\text{-}SG}$ is the distance between the SG in Cys169 of the nitrilase and cyano group (C8) in substrates. $D_{N1\text{-}HZ}$ is the distance between the HZ atom in Lys135 of the nitrilase and cyano group (N1) in substrates. [b] the average value during the 10 ns molecular dynamics performed using the standard AMBER99 force field.

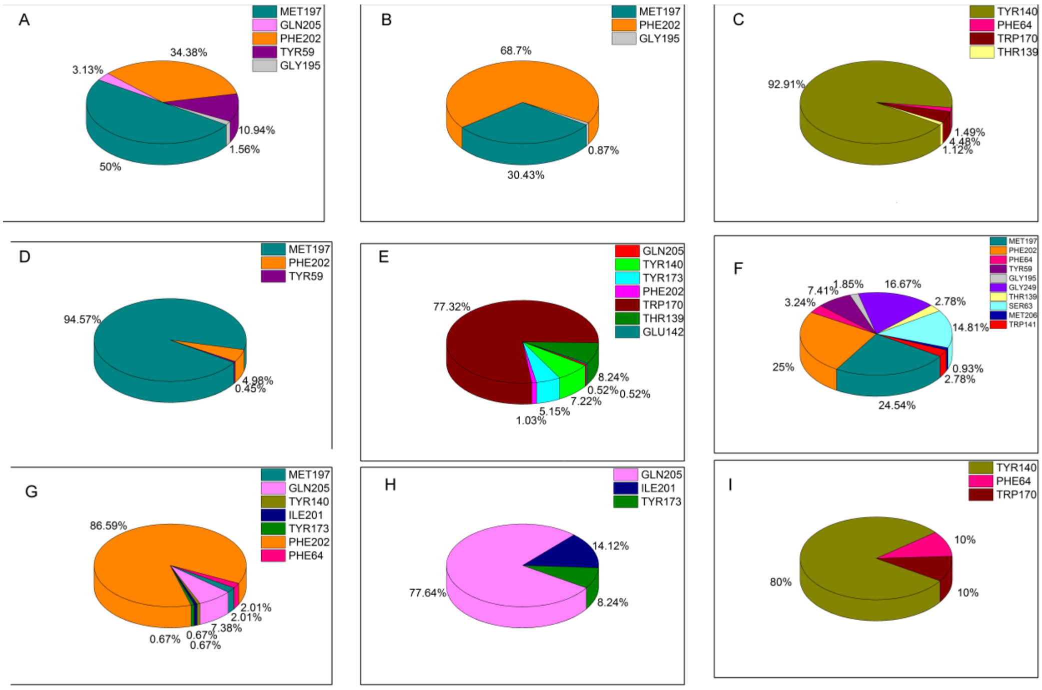

Figure S4. The profile of halogen bonds formed between substrates and different residues in the nitrilase and mutants during the 10 ns dynamic simulation performed using the standard AMBER99 force field. (A) WT and 1c (B) WT and 1a; (C) WT and 1b (D) H141W and 1c (E) H141W and 1a; (F) H141W and 1b (G) T54Y and 1c (H) T54Y and 1a; (I) T54Y and 1b

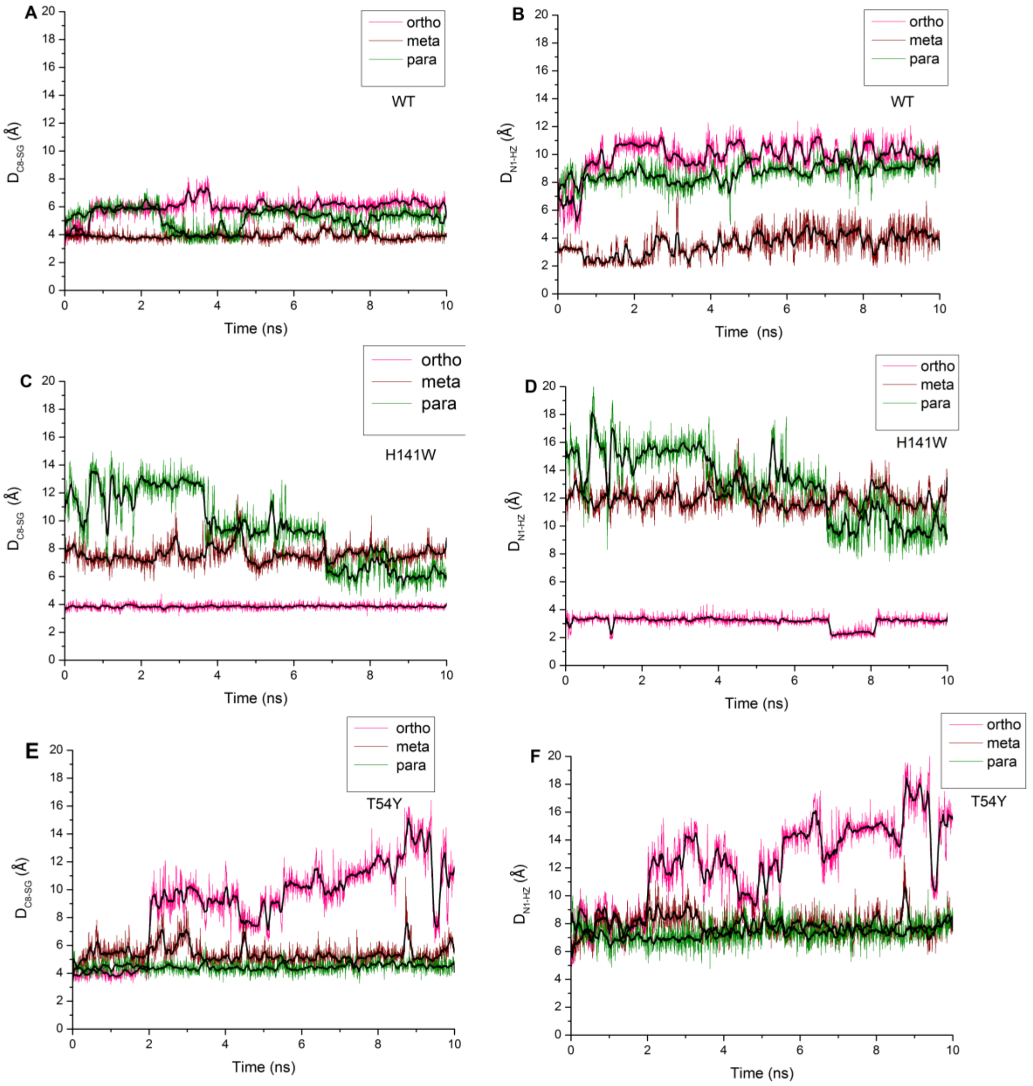

Figure S5. The $D_{N1\text{-}HZ1}$ and $D_{C8\text{-}SG}$ in the complexes of WT, H141W and T54Y with 1a, 1c and 1b during 10 ns MD simulation performed using the standard AMBER99 force field.

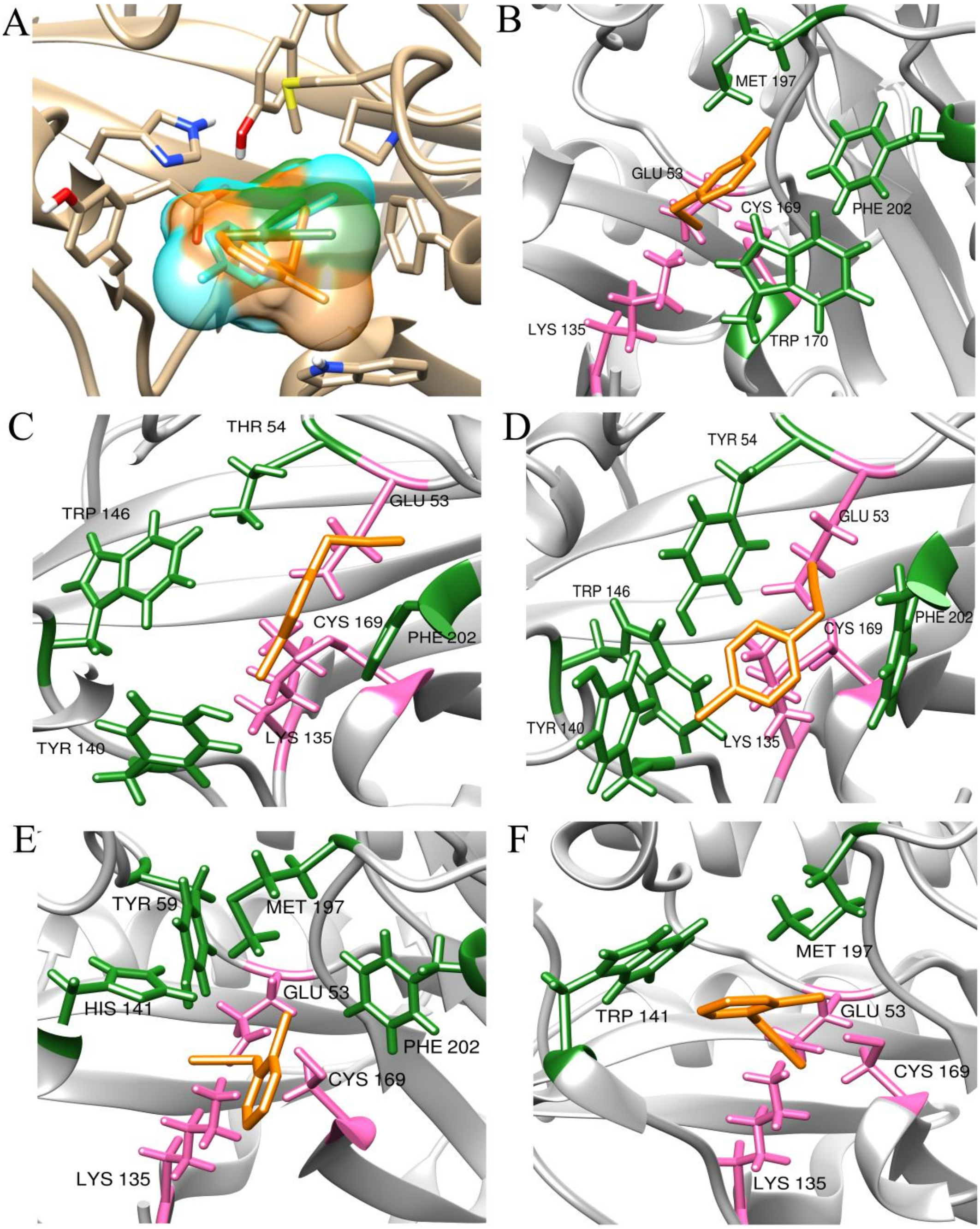

Figure.S6 The surface of 1a, 1b and 1c in the binding pocket of WT. 1a, 1b and 1c are shown in green, cyan and orange (A). And the halogen bonds and aromatic interactions in complexes: WT-1a (B), WT-1b (C), T54Y-1b (D), WT-1c (E), H141W-1c (F). The 10ns MD simulation was performed using the standard AMBER99 force field.The catalytic triad residues Cys169-Glu53-Lys135, substrates, residues contribute to halogen bonds and residues contributed to aromatic interactions are shown in pink, orange, and green, respectively.
